# HIV-Associated Neurocognitive Disorders: The Relationship of HIV Infection with Physical and Social Comorbidities

**DOI:** 10.1155/2015/641913

**Published:** 2015-03-01

**Authors:** Ellen M. Tedaldi, Nancy L. Minniti, Tracy Fischer

**Affiliations:** ^1^Department of Medicine, Temple University School of Medicine, Philadelphia, PA 19140, USA; ^2^Department of Physical Medicine and Rehabilitation, Temple University Hospital, Philadelphia, PA 19140, USA; ^3^Department of Neuroscience, Temple University School of Medicine, Philadelphia, PA 19140, USA

## Abstract

The prevalence of HIV (human immunodeficiency virus) associated neurocognitive disorders (HAND) will undoubtedly increase with the improved longevity of HIV-infected persons. HIV infection, itself, as well as multiple physiologic and psychosocial factors can contribute to cognitive impairment and neurologic complications. These comorbidities confound the diagnosis, assessment, and interventions for neurocognitive disorders. In this review, we discuss the role of several key comorbid factors that may contribute significantly to the development and progression of HIV-related neurocognitive impairment, as well as the current status of diagnostic strategies aimed at identifying HIV-infected individuals with impaired cognition and future research priorities and challenges.

## 1. Introduction

HIV-1 infection often results in varying degrees of neurocognitive dysfunction, ranging from mild impairment to frank dementia, collectively termed HIV-associated neurocognitive disorders (HAND) [1]. In its latest revision, the Diagnostic and Statistical Manual- (DSM-) 5 categorizes mild and major neurocognitive disorders on the basis of presumed etiology, association with behavioral disturbances, and degree of severity [2]. Although infection with the virus itself is included in the list of possible explanations for cognitive dysfunction among HIV-infected individuals, in clinical practice there remains the ongoing challenge of establishing the impact of the virus as the source of impairment, in relation to a variety of numerous clinical, social, and psychological factors that may contribute to HAND [3–14]. For example, neurocognitive impairment is often seen in cardiovascular disease and sleep disorders, in both HIV negative and positive persons [15–22]. With the addition of successful combination antiretroviral therapy (cART) and patients living longer with HIV, these comorbidities are highly prevalent in the aging HIV population [23–28]. There are diagnostic issues related to the sensitivity to detect degrees of impairment especially in the ethnically and educationally diverse HIV populations of the current US epidemic where normative data may be lacking [10, 29–38]. HAND may be the diagnosis of exclusion as there are no definitive biomarkers or “gold standard” assays to confirm this diagnosis [39].

The spectrum of neurocognitive disorders has evolved in the contemporary treatment era [11, 40–45]. The Frascati criteria, based on a consensus panel convened by the National Institute of Mental Health (NIMH) and National Institute of Neurological Diseases and Stroke (NINDS) in 2007, are the basis for distinguishing the categories of HAND: asymptomatic neurocognitive impairment (ANI), minor neurocognitive disorder (MND), and HIV-associated dementia (HIV-D) [1]. As widely reported elsewhere, there has been a significant decline in HIV-D but a higher persistence of the minor categories, with frequently cited percentages of 20% for HIV-D and as much as 50% for minor cognitive disorders [11, 46].

## 2. Neuropsychological Assessment

Tools for assessing neurocognitive disorders include a variety of established, as well as emerging, technologies that include neurocognitive testing batteries, neuroimaging, and biomarkers [29, 36, 45, 47–56]. This review will highlight some of the challenges and controversies that make assessment and diagnosis of neurocognitive disorders in HIV-infected persons a complex process that will necessitate revised strategies.

It has been established that HIV infection impairs cognition through a variety of mechanisms including CNS entry of the HIV-1 infected monocyte derived macrophages that cross the blood-brain barrier to result in a cascade of events including release of cytokines and viral products that can disrupt endothelial barriers and affect neuronal pathways [57–59]. Chronic inflammation and ongoing HIV replication that persists despite peripheral HIV suppression from antiretroviral therapy may compartmentalize in various areas of the CNS leading to dysregulation of neuronal pathways [60–63].

**Figure 1:**
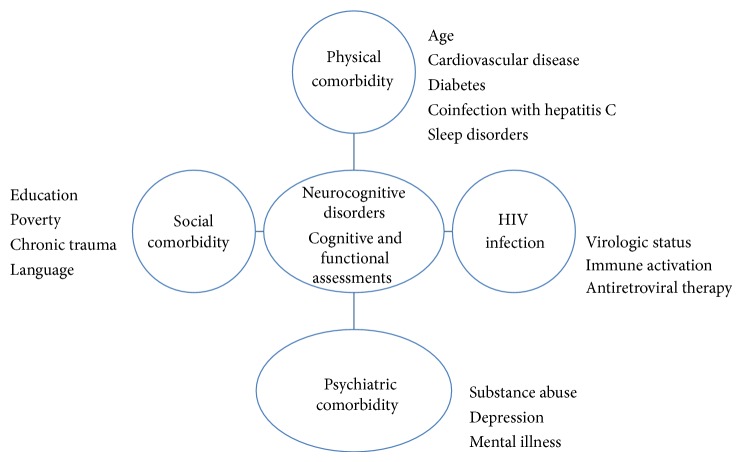
Neurocognitive disorders: role of HIV infection, comorbidities, and assessments.

The neurocognitive profile of HAND has included deficits in psychomotor slowing, impaired episodic memory, prospective memory, attention and working memory, and verbal fluency [11, 42, 45, 64]. In addition to the immunologic and virologic status of infected persons, coexisting conditions that contribute to cognitive status include medical conditions and/or psychiatric disorders, substance abuse, and education/literacy (Figure 1) [12, 15, 33, 65–73]. The dilemma for clinicians and scientists is to delineate the role of the virus from other influences on neurocognition. The standard neuropsychological test battery can be labor intensive and requires trained personnel to administer and evaluate it. While several rapid screening instruments have been developed that can detect cognitive decline in HIV-infected persons, there is a paucity of comparative normative data in matched non-HIV populations [33, 47, 51, 53, 55, 56, 74–83]. Demographically adjusted normative data (i.e., adjustments to scores based on the subject's age, education, and/or race) are essential for accurately assessing HAND. In many cases, however, these data are lacking when comparison groups are needed for individuals of low socioeconomic status (SES) or of a racial or ethnic minority. Most neuropsychological tests are developed and normed on members of the majority culture, offering little to no information regarding test performance of members of racial/ethnic minorities, those with limited or no formal education and poor literacy, or those who grew up in very different circumstances (e.g., rural environments). Clinicians are often left with poorly matched normative groups and subsequent limitations in interpreting scores. This lack of appropriate normative information can falsely classify members of a minority culture as having cognitive decline or dementia. Inappropriate diagnosis of HAND can have profound negative implications, leading to inappropriate medical intervention, reduced self-esteem, negative financial repercussions, reduced functional independence, and misdirection of future planning. Functional capacity assessment also has limitations in the neurocognitive evaluation toolbox. These are often self-reported data, which have inherent variability issues, and some instruments are not culturally or educationally suited to administer in diverse population [49, 84–87].

## 3. Social Comorbidities Effecting Cognition

### 3.1. Education

The influence of demographic variables such as age, education, ethnicity, and sex on neuropsychological test results has been widely observed and their influence is significantly related to most cognitive domains in varying degrees [88]. Despite this awareness, many neuropsychological tests do not stratify their data based on these variables and, as such, the effects of demographic characteristics on particular tests are unknown. Even when tests are stratified by age or education, little information is available on how those from disadvantaged backgrounds or individuals with low literacy may perform, and recent investigations suggest that these variables are critical to understanding neurocognitive test performance. For example, it has been observed that African Americans often obtain lower scores on a broad range of cognitive tasks in comparison to their non-Hispanic white counterparts, although the reasons for these discrepancies are not entirely clear [89–91]. Educational attainment has been used as a means for controlling these differences as there are often variations in years of education between African-Americans and Caucasians. Investigators will use covariance or matching procedures as a means of equating racial groups on years of education before comparing test performance. One hypothesis put forth to account for these differences is disparities in educational quality that exist across racial and ethnic groups [92–94]. Numerous studies have indicated that reading ability is a better predictor of cognitive performance than years of education in African Americans and individuals of disadvantaged socioeconomic backgrounds [30, 93, 95]. In a study of HIV-infected participants, racial and ethnic minorities had lower levels of literacy than nonminority subjects. Those with the highest discrepancy between reading ability and educational attainment had worse neuropsychological performance, while minority status was not associated with test performance, while minority status was not associated with test performance [96]. This demonstrates that, for some cohorts of HIV+ subjects, literacy is a better marker of educational experience and may account for observed differences in racial groups [97]. Therefore, individuals with low levels of literacy require special consideration when interpreting test results, and particular care should be taken to avoid overpathologizing patients with HIV. It should be noted that low levels of literacy and/or limited educational opportunity are also associated with more rapid age-related cognitive and functional decline and higher rates of dementia in general and should be considered as a possible contributory or confounding factor in the diagnosis of HAND [93]. These findings underscore the need for appropriate normative groups in the assessment of HAND, as HIV disproportionately affects ethnic minorities and those from disadvantaged educational backgrounds.

### 3.2. Poverty and Excessive Exposure to Trauma

In addition to education, other “social comorbidities” that may impact cognition among HIV-infected individuals include poverty and chronic exposure to trauma [98–100], which may adversely affect the “cognitive reserve” of individuals subject to these conditions prior to contracting HIV. Cognitive reserve refers to the development of extensive neuronal connections that can protect neurons when they are subjected to injury through oxidative stress or inflammation [101]. The amount of reserve can be influenced by positive (education, new task) or negative neuroplasticity (poverty, isolation) [81, 100–104]. For HIV-infected populations, which are overrepresented in impoverished communities, chronic exposure to limited resources, violence, or trauma can erode neurocognitive reserves with resultant impairment that may be additive with that resulting from HIV and/or other conditions [49, 85, 86, 100, 105–107].

## 4. Physical Comorbidities Effecting Cognition

### 4.1. Aging

A multitude of physical comorbidities have been shown to effect cognition in general populations: age, cardiovascular/metabolic disease, sleep disorders, and coinfections, such as hepatitis C virus (HCV) [17, 19, 20, 67, 108–114]. Age is a major risk factor for dementia, with 0.8% of persons being 60–65 years old in the US reported to have dementia from any etiology [115]. The US HIV population is poised, therefore, to see more cases of dementia; 19% of the current HIV population and 5% of new HIV diagnoses are people over 55 years of age [25, 116]. The HIV associated non-AIDS medical conditions. the HIV associated Non-AIDS conditions (HANA) and the aging of the HIV population will contribute to the expansion of the cognitively impaired population of older individuals.

Two long-standing cohorts of HIV positive and HIV negative men and women, the Multicenter AIDS Cohort (MACS) and the Women's HIV Interagency Study (WIHS), respectively, provide unique longitudinal data that can elucidate the relative contribution of HIV infection to mortality. In early initiators of HIV therapy and the HIV negative cohorts, the risk for non-AIDS related mortality was comparable [117]. As longevity with HIV infection improves with earlier treatment, the neurocognitive disorder burden will not only increase but may also evolve differently with longer durations of immune activation [40, 118–121]. The pathophysiology of age-related neurocognitive disorders may be similar to and synergistic with those in HIV, including chronic inflammation, oxidative stress, and immune senescence. It has been noted that the clinical presentation of HIV-D, traditionally a subcortical phenomenon, is now similar to the cortical dementias, such as Alzheimer's disease [45].

### 4.2. Cardiovascular Disease

Cardiovascular disease (CVD) is a major contributor to neurocognitive disorders in the general population [22, 122, 123]. The development of vascular cognitive impairment involves interplay of traditional cardiovascular factors, such as hypertension, diabetes and hyperlipidemia, and behavioral ones, such as obesity and sedentary life style [3, 110, 124]. Mechanistically, they cause inflammation and oxidative stress to blood vessels and may cause cerebral hypoxia-ischemia and small vessel endothelium dysfunction. HIV infection, itself, can share these common pathways and is associated with increased vascular morbidity through elevated levels of inflammatory and procoagulant factors, atherogenesis, and endothelial alterations [6, 125–127].

In addition, the role of antiretroviral therapy, especially protease inhibitors, has been implicated in the development of metabolic syndrome with insulin resistance, dyslipidemia, and hypertension [26, 28, 128–131]. If obesity and cigarette smoking are included in the risk profiling for vascular disease, HIV populations are population-matched in obesity rates and exceed peer groups in smoking frequency [20, 38, 132].

Reports from cohort studies and clinical trials indicate that neurocognitive performance of HIV patients with CVD risk factors is impaired [3, 10, 103, 131, 133–135]. HIV is disproportionately represented among African Americans and Latinos, who also bear a greater burden of CVD, as compared to other ethnic groups [136–138]. Compounded with the unfortunate realities of later presentation for care and lower rates of virologic control, the risk for vascular-associated neurocognitive disorders in these populations is greater [139–141]. The compelling clinical issue is whether CVD risk modification with optimal virologic control and metabolically favorable antiretroviral therapy will be sufficient when some of the pathogenetic mechanisms such as immune activation persist.

### 4.3. Sleep Disorders

Sleep disorders, especially obstructive sleep apnea, are implicated in neurocognitive dysfunction in adults [17, 18, 111, 112]. The prevalence of sleep-related disorders is common in the US and there are several reports on the prevalence in HIV populations [24, 142–146]. The deleterious effects of sleep deregulation on neurocognitive performance can result from obstructive sleep apnea, medication, or coexisting depression [15]. The literature on sleep and HIV and neurocognition had focused on the CNS effects of efavirenz but this effect on sleep and neurocognition can be variable in duration [147]. Sleep disorders are also associated with other medical conditions including metabolic syndrome and cardiovascular disease [148–152]. The synergistic effect of sleep disorders and cardiovascular and metabolic complications on neurocognition in HIV patients has not been reported extensively. Our group is currently surveying a diverse group of English and Spanish speaking patients to determine the prevalence of sleep disorders and relationship to a variety of immunologic, virologic, and clinical outcomes. Whether the correction or improvement in the underlying sleep dysfunction will improve neurocognitive and functional outcomes is an area of ongoing investigation.

### 4.4. HCV Coinfection

Depending on the HIV population, coinfection with HCV can be found in 25% of HIV populations overall and upwards of 75–85% in high-risk groups, such as intravenous drug users [153–155]. Cognitive impairment has been noted in HCV in higher proportions than HIV infection alone, although not consistently [7, 67, 70, 156–159]. HCV shares similar pathways of immune activation and inflammation that can affect neurocognitive function [60]. HCV/HIV coinfection is often present in individuals with other confounders of cognitive impairment such as psychiatric disorders and substance abuse [65]. With the advent of more effective therapeutic options for HCV, successful eradication of HCV may have a major salutary effect on neurocognition [160]. Improvement, however, will depend on successful control of other comorbidities, including cessation of drug use and engagement in medical care.

### 4.5. Illicit Drug Use

The prevalence of mental illness and substance abuse among HIV-infected patients is greater than the general population and is a significant risk factors for HIV acquisition [12, 161–165]. This population often has neurocognitive sequelae from a variety of illicit drugs, including cocaine, opiates, methamphetamine, and marijuana. The effect of illicit drugs on neurocognitive impairment has been well documented [163, 166–170] and negatively impacts the treatment of these individuals. The pathogenetic mechanisms of HAND are believed to involve increased oxidative stress and permeability of the blood-brain barrier (BBB) to neurotoxic factors, including HIV viral proteins, tat, and gp120. Given the risk for nonadherence to antiretroviral therapy among drug users, the consistency of viral suppression may be less, leading to frequent episodes of viremia and uncontrolled virus replication [60, 171–173].

### 4.6. Depression and Mental Illness

Depression is common in persons living with chronic disease including HIV and is associated with several adverse outcomes including medication nonadherence, impaired functional status, neurocognitive impairment, and mortality [33, 174–180]. Clinicians do have well established brief screening instruments for depression available to them that can be utilized repeatedly for assessment [181–183]. Neurocognitive assessment generally includes an evaluation for depression since cognitive impairment is common with this disorder and may be improved with therapeutic interventions including treatment with antiretroviral therapy [177].

Other psychiatric disorders such as schizophrenia or bipolar disease are often seen in a subset of HIV-infected persons who have substance abuse and tenuous social and economic support. They are at extremely high risk for medication nonadherence and may have the “perfect storm” of cofactors that result in neurocognitive impairment [61, 66].

### 4.7. Chronic Systemic Inflammation in HAND Pathogenesis

It remains unclear why some individuals develop a pathological correlate of the most severe form of HAND, HIV-D, and HIV encephalitis (HIVE), while the majority of infected persons do not. Yet, understanding the pathogenesis of HIVE may provide key insights into the milder forms. In HIVE, activated infected and noninfected macrophages (MΦs) and microglia play a prominent role in the development and progression of disease through the secretion of chemokines, cytokines, and other inflammatory mediators that are directly or indirectly neurotoxic. Additionally, infected MΦs and microglia may spread virus to noninfected cells and release viral proteins that contribute to prolonged brain inflammation and neurotoxicity (for review, please see [59, 184, 185]). Together, this suggests an important role of both the virus and inflammation in HIVE pathogenesis. A recent immunohistological study of brain tissue from HIV-infected persons without encephalitis but with varying degrees of neurocognitive impairment shows phenotypical and morphological evidence of inflammation, in the absence of virus production, which is similar to, but less severe than, that seen in HIVE [63]. These findings support the notion that HAND is a continuum of the same disease process and demonstrate that inflammation, rather than virus production in the brain, bridges the milder and severe forms. These studies also reveal perivascular astrocyte activation in cortical grey matter of brain from HIV-infected persons without encephalitis that does not extend into the brain parenchyma, suggesting events at the BBB precede parenchymal involvement [63]. Interestingly, while cART appears to have significantly reduced the incidence of HIV-D, milder forms of HAND have become an increasing issue [41]. This is likely due to the ability of cART to slow the disease process, while the underlying mechanism driving the development and progression of HAND (i.e., chronic immune activation) continue.

Although the brain is often referred to as an “immune privileged” site, there is increasing awareness of the significant communication between the brain and peripheral immune system. Systemic administration of lipopolysaccharide (LPS) in rat and mouse models promotes neuroinflammation and neuronal loss [62, 186] and has been shown to exacerbate neuronal loss in animal models of neurodegenerative disease [187]. In the setting of chronic inflammatory conditions, such as HIV infection, amplified and/or prolonged immune signals from the periphery to the brain may have profound consequences on the diseased or healthy brain. This is evidenced by relapses in multiple sclerosis (MS) symptoms following systemic infection [188, 189] and diminished cognitive capacity seen in individuals with rheumatoid arthritis (RA) or systemic lupus erythematosus [190, 191], as well as other conditions associated with impaired immunity, such as ageing.

Communication pathways from the periphery to the brain are well reviewed [192–194] and involve cytokines commonly seen in systemic infections and inflammatory conditions, including interleukin- (IL-) 1*β*, IL-6, and tumor necrosis alpha (TNF*α*), which are also elevated in HIV infection. As put forth by Perry and colleagues in (non-HIV) diseased and aged brain [193], microglia in brain of HIV-infected persons may be “primed” to quickly switch to an activated phenotype in response to inflammatory stimuli from the periphery. This may occur during the acute phase of infection, which is associated with significant immune activation and cytokine production [195]. It is during this stage of infection that virus first appears in the brain; however, it remains unclear whether HIV infection is established in the brain at this time [196]. Once primed, microglia may launch a sustained and/or exaggerated immune response to inflammatory mediators from the periphery that impairs the function of neurons and other glia, resulting in cognitive dysfunction.

### 4.8. Microbial Translocation as a Source of Chronic Inflammation in HIV Infection

Microbial translocation, which refers to the passage of live bacteria and/or bacterial products (e.g., LPS) across an anatomically intact intestinal barrier, has been suggested to be a major factor in HIV-related chronic immune activation and may contribute to HAND pathogenesis. Also referred to as “leaky gut,” microbial translocation can result from reduced host immunity and/or increased permeability of the intestinal lining and has been shown in a number of infectious and noninfectious chronic inflammatory diseases, including celiac disease, inflammatory bowel disease, and hepatitis B and hepatitis C [197–199]. Evidence of microbial translocation, including elevated plasma LPS, LPS-binding protein (LBP), soluble CD14 (sCD14), and bacterial 16S ribosomal DNA (rDNA), has been reported by a number of groups in HIV-infected human subjects and SIV infected rhesus macaques [200–205]. While the pathogenic mechanisms involved in the loss in integrity of the mucosal barrier in HIV remain unclear, early mucosal CD4 T cell depletion and HIV-related enteropathy are believed to play a prominent role [206–209]. Seminal work by Brenchley et al. suggested that microbial translocation directly contributes to systemic innate and adaptive immune activation during chronic HIV infection and may affect the progression rate of AIDS [208]. Indeed, increased plasma 16S rDNA has been associated with greater T cell activation in HIV-infected subjects and reduced CD4^+^ T cell restoration with cART [201]. In addition, plasma LPS and soluble CD14 (sCD14) levels, a marker associated with monocyte stimulation by LPS, are reported to be elevated in patients with HIV-D, as compared to those with normal cognition [203]. In this same study, the percent of frequency CD16^+^ monocytes and plasma LPS was shown to decrease in patients taking cART for at least 8 weeks [203].

### 4.9. CD16^+^ Monocytes as a Potential Biomarker for Neurocognitive Dysfunction

There is intense interest in determining the role and specificity of biomarkers that may correlate with HAND or other neurocognitive disorders. Although these assays are not currently used in routine clinical practice, they may function as a component of a diagnostic algorithm to diagnose or predict cognitive impairment [48, 210–212]. Previously, the CD16^+^ monocyte subset has been shown by a number of groups to be elevated in HIV infection [213–216], with an even greater frequency seen in patients with HIV-D [217]. A more profound relationship with HIV disease progression is suggested; however, when the CD16^+^ monocyte subset is further subfractionated by CD163 expression, where a positive correlation with CD16^+^/CD163^+^ monocyte frequency and viral load, as well as CD4^+^ T cell loss in individuals with 450 cells/*μ*L or less, has been observed [216]. Interestingly, these cells are phenotypically similar to MΦs that accumulate in HIVE brain, comprise perivascular cuffs and nodular lesions, and serve as the principal reservoir of productive virus in brain [218, 219]. Further, this increase in total brain MΦs in HIVE appears to be due to trafficking of monocytes/MΦs from the periphery, rather than microglial proliferation [220]. Additional studies are needed to determine whether the frequency of these monocyte subsets correlates with cognitive performance and whether CD16^+^ or CD16^+^/CD163^+^ monocyte frequency can serve as a viable biomarker for HIV disease progression and neurocognitive impairment.

## 5. Conclusions

Neurocognitive disorders are evolving in the HIV population with ageing and a variety of comorbid physical, social, and psychological factors, creating a complex interplay in pathogenesis, clinical presentation, and outcomes. Important areas for research include developing validated rapid screening instruments with population-specific normative data for identifying cognitive impairment in clinical practice, advancing our current understanding of HAND pathogenesis, which will allow for the identification of potential biomarkers and/or targets for therapeutic intervention, and identifying and or developing behavioral interventions to prevent or treat neurocognitive disorders.

In screening for HAND, Kamminga et al. recommended several criteria for optimal screening procedures that include targeting outcome measures that are more predominant in the cART era, which are compared to the comprehensive neuropsychiatric battery in non-HIV populations, as well as instruments that have normative data in diverse populations among other criteria [34]. One important criterion is that these screens can be used in the clinical setting where resources limit more time intensive approaches. In the context of multimorbidity and HAND, the assessment and treatment challenges of an HIV-infected patient are several:

1. the diagnosis and treatment of cofactors for HAND, such as hypertension, diabetes, obesity, and coinfection: each entity has its own particular and shared clinical and lifestyle approaches, and control and management of these significant correlates of cognition in the context of polypharmacy and drug-drug interactions with antiretroviral therapy are a challenge for adherence;
2. behavioral and mental health interventions to address concurrent drug use and psychiatric disease that can be integrated into the clinical settings;
3. functional assessments for frailty and cognitive impairment that will identify earlier stages for intervention;
4. earlier detection of HIV and engagement in care: the chronic neurocognitive sequelae of HIV disease and the other comorbidities will be minimized with successful long-term adherence to therapy and to a comprehensive treatment approach.

The social comorbidities that affect HAND and other neurocognitive disorders will continue to be a particularly difficult factor to modify as HIV infection predominates in under resourced and economically marginalized communities. Translational research approaches for neurocognitive disorders will need to focus on these intersections of multiple physical and social comorbidities in a global assessment schema. The models for chronic disease management will be useful for those living with HIV/AIDS.
